# Pathways Linking the Big Five to Psychological Distress: Exploring the Mediating Roles of Stress Mindset and Coping Flexibility

**DOI:** 10.3390/jcm11092272

**Published:** 2022-04-19

**Authors:** Luxi Chen, Li Qu, Ryan Y. Hong

**Affiliations:** 1 Centre for Family and Population Research, National University of Singapore, Singapore 117570, Singapore; 2 School of Social Science, Nanyang Technological University, Singapore 639818, Singapore; quli@ntu.edu.sg; 3 Department of Psychology, National University of Singapore, Singapore 117570, Singapore; ryan.hong@nus.edu.sg

**Keywords:** Big Five, coping flexibility, stress mindset, threat, challenge, psychological distress

## Abstract

Personality affects the vulnerability to the emotional symptoms of depression and anxiety. This study investigated whether stress mindset (general belief about the nature of stress) and coping flexibility (the ability to terminate ineffective coping strategies and adopt alternative ones) mediate the relations of the Big Five personality traits to psychological distress. A total of 260 undergraduate students (60.4% female) in Singapore completed self-reported questionnaires. A series of path analyses was performed. Firstly, a dual-pathway model of stress coping was established, which consisted of (a) a stress–threat–distress pathway where a stress-is-a-threat mindset mediated the association between stressful experiences and psychological distress and (b) a challenge–flexibility–enhancement pathway where coping flexibility mediated the relation of a stress-is-a-challenge mindset to a lower level of psychological distress, without being influenced by stressful experiences. Furthermore, Neuroticism was associated with the stress–threat–distress pathway, with stressful experiences and a stress-is-a-treat mindset mediating the relation of Neuroticism to psychological distress. Conscientiousness was associated with the challenge–flexibility–enhancement pathway, with a stress-is-a-challenge mindset and coping flexibility mediating the relation of Conscientiousness to less psychological distress. Extraversion, Agreeableness, and Openness were directly associated with greater coping flexibility. The findings enrich the literature on personality and stress coping and inform future interventions to promote mental health.

## 1. Introduction

Psychological distress, incorporating anxiety, depression, and other emotional symptoms such as stress or tension, is an important indicator of mental health [1]. The increasing prevalence of mental health issues over the past decades and its impacts on a wide range of social and economic implications have highlighted the importance of preventing mental disorders and promoting mental health [2]. Understanding the mechanisms underlying psychological distress is a critical step prior to designing effective prevention and intervention programs. 

Different personality traits act as risk or protective factors of mental health by affecting one’s exposure to stressful events, stress appraisals, as well as coping strategies [3,4,5]. According to the transactional model of stress coping [6,7] and the appraisal model of emotion [8,9], stress appraisals (referred to as the evaluation of personal competence to meet the situational demands) and coping strategies (referred to as cognitive and behavior effort aiming to manage external or internal demands) mediate the relationship between the experiences of stressful events and emotional symptoms. Recently, increasing attention has been paid to *stress mindset*, which is independent of any situation and defined as a meta-cognitive belief about the nature of stress as enhancing or debilitating [10,11]. The impact of perceived stress on mental health issues can be aggravated by a stress-is-debilitating mindset but mitigated by a stress-is-enhancing mindset [12]. Moreover, a growing body of research has shown that, rather than using a particular strategy across situations, having the ability to monitor and modify coping strategies to meet the demands of various situations, defined as coping flexibility, can predict fewer depressive and anxiety symptoms [13,14,15]. Despite this large body of research on stress coping, very little research has investigated the roles that stress mindset and coping flexibility play in the relations of different personality traits to mental health. The current research sought to explore whether and how stressful experiences, stress mindset, and coping flexibility work with one another to act as the explanatory mechanism underlying the influence of personality on psychological distress. 

Stress mindset reflects the extent to which people believe stress generally has enhancing outcomes related to performance, learning, personal growth, health, and well-being (referred to as a stress-is-enhancing mindset) or debilitating outcomes (referred to as a stress-is-a-debilitating mindset) [10,11]. In the present study, we classified stress mindset as a *stress-is-a-challenge mindset* (to interpret the nature of stress as opportunities for personal growth and gain) and a *stress-is-a-threat mindset* (to interpret the nature of stress as a damage or loss) [16]. Stress mindset influences cognitive, affective, and coping responses across different types of stressors and then determines people’s downstream performance, decisions, and health outcomes [10,11,16]. In particular, a threat/debilitating mindset is associated with a higher risk of depression, whereas a challenge/enhancing mindset can mitigate the impacts of stressful experiences on depressive symptoms [12,16]. 

Derived from the transactional model of stress coping, Kato’s dual-process theory defined coping flexibility as the ability to evaluate coping strategies, relinquish an ineffective strategy, and select a more appropriate one [17,18]. People with a rigid coping style usually exhibit more depressive and anxiety symptoms, whilst having greater flexibility to attune strategies to changing situations can promote psychological adjustment [13,14,15,19,20,21,22]. Moreover, the literature suggests that coping flexibility interplays with stress interpretational styles to determine emotional disorders such as anxiety and depression [23]. Some recent studies found that coping flexibility was positively associated with challenge appraisal [24] and a stress-is-a-challenge mindset [25]. The interrelationships among stressful experiences, stress mindset, coping flexibility, and psychological distress, however, are still unclear. Hence, the first aim of the current research was to explore the roles of stress mindset and coping flexibility in the relationship between stressful experiences and psychological distress.

Furthermore, the Big Five personality traits influence mental health through exposure to stress, stress appraisal, and coping strategies [3,4,5]. The Big Five dimensions of personality include Neuroticism, Conscientiousness, Extraversion, Agreeableness, and Openness to experience [26,27]. Little is known about whether different stress mindsets and coping flexibility also explain the different effects of the Big Five personality traits on mental health. To fill this gap, we aimed to examine the mediating roles of stress mindset and coping flexibility in the relations of the Big Five to psychological distress.

Neuroticism is characterized by negative emotionality, physiological reactivity to stress, and behavioral inhibition [27,28]. People higher on Neuroticism tend to perceive more experiences of stressful events, interpret stress as more threatening, and engage in more dysfunctional coping strategies (e.g., self-blaming, disengagement, and denial), all of which are associated with more emotional symptoms such as depression and anxiety [4,29,30,31,32]. Given that people high on Neuroticism tend to engage in threat appraisal in different situations, they may hold a stress-is-a-threat mindset to interpret stress as a loss, harm or damage, in general. In addition, when preoccupied with negative affect, negative thoughts, and avoidance coping, high-Neuroticism individuals may not actively reflect on their coping strategies or modify these strategies in order to adapt to the changing situations. In sum, the association between Neuroticism and psychological distress may be mediated by the experiences of stressful events, a stress-is-a-threat mindset, and inflexible coping.

Conscientiousness embodies deliberation, self-regulation, impulse control, and achievement orientation [27,28]. People high on Conscientiousness tend to perceive less environmental threats and have a wide range of functional coping strategies such as problem solving, emotional regulation, and cognitive restructuring [4,33,34,35,36]. Conscientious persons usually engage in positive stress appraisal, so they may also hold a general belief that stress provides opportunities for enhancing outcomes. Having a functional coping repertoire allows them to re-cope with different situations by selecting the adaptive strategies in a flexible manner. The achievement orientation and good planning skills also facilitate high-Conscientiousness people to continuously evaluate their coping strategies and modify their coping strategies to meet the situational demands. Hence, it is reasonable to expect that a stress-is-a-challenge mindset and coping flexibility may mediate the relation of Conscientiousness to a lower level of psychological distress.

Agreeableness involves trust, altruism, compliance, and prosocial orientation toward others [27,28]. People high on Agreeableness tend to engage more adaptive coping strategies such as seeking instrumental and emotional support [37]. With the ability to conform to different social environments, high-Agreeableness individuals may be able to actively modify coping strategies according to the situational demands, so as to adapt to changing situations. Thus, coping flexibility may be one important capacity that explains the association between Agreeableness and fewer mental health issues.

Extraversion is characterized by positive emotionality, sociability, assertiveness, high activity levels, and sensitivity to reward [27,28]. People high on Extraversion usually perceive adequate coping resources to deal with stress [4,38]. They are also equipped with proactive coping strategies such as problem solving, support seeking, and cognitive restructuring [39], which can form a functional coping repertoire. The assertiveness and high activity levels may facilitate extroverted individuals to rapidly judge the effectiveness of a coping strategy, terminate the ineffective one, and select an alternative one. We expected that Extraversion would be positively associated with coping flexibility.

Openness to experience signifies the tendency to be creative, curious, flexible, imaginative, and it involves a range of intellectual interests [27,28]. People who are open to new experience usually perceive less threats or fewer demands and use humor to cope with stress [4,40,41]. Their creativity, flexibility, and intellectual interests may facilitate the abandonment of coping strategies that failed to manage the stressor and the re-selection of a more appropriate one. Thus, we expected that Openness may be related to greater coping flexibility. 

Taken together, the current study sought to examine the pathways linking the Big Five to psychological distress by focusing on the mediating roles of stress mindset and coping flexibility. The following hypotheses were derived from the literature:

**Hypothesis** **1** **(H1).** *Neuroticism is associated with more stressful experiences, a stress-is-a-threat mindset, a lower level of coping flexibility, and greater psychological distress*.

**Hypothesis** **2** **(H2).** *Conscientiousness, Agreeableness, Extraversion, and Openness are associated with a stress-is-a-challenge mindset, greater coping flexibility, and a lower level of psychological distress*.

**Hypothesis** **3** **(H3).** *A stress-is-a-threat mindset mediates the relation of stressful experiences to greater psychological distress*.

**Hypothesis** **4** **(H4).** *Coping flexibility further mediates the relationship between stress mindset and psychological distress, and to be more specific*.

**Hypothesis** **4a** **(H4a).** *Low coping flexibility mediates the association between a stress-is-a-threat mindset and greater psychological distress*.

**Hypothesis** **4b** **(H4b).** *Coping flexibility mediates the association between a stress-is-a-challenge mindset and a lower level of psychological distress*.

## 2. Materials and Methods

### 2.1. Participants and Procedure

A total of 260 undergraduate students (60.4% women; *M_age_* = 21.4, *SD_age_* = 1.70, range: 19–26 years) who were not psychology majors were recruited from the research pool at the Nanyang Technological University, Singapore. These participants majored in different subjects and were enrolled in the Introduction to Psychology course, which required them to earn 10 research credits in the semester through participation in psychological studies. Eligible students selected and signed up for the studies that they were interested in from the available ones in the research pool system. Each participant received 3 research credits for taking part in the current study. G*Power 3.1 [42] was used to confirm that we can achieve statistical power 90% with the current sample size, based on the smallest meaningful effect size for interpretation (*r* = 0.20). Ethical approval was obtained from the university’s Institutional Review Board.

Participants completed the study individually during one single-session administration in a quiet laboratory room. After providing informed consent, all participants responded to a set of validated scales on stressful experiences, the Big Five personality traits, stress mindset, coping flexibility, and psychological distress. All measures were administered in English.

### 2.2. Materials

#### 2.2.1. Stressful Experiences

The 49-item Inventory of College Students’ Recent Life Experiences (ICSRLE) Scale [43] was adopted to measure university students’ stressful experiences, such as academic alienation (e.g., “*Struggling to meet your own academic standards*”), time pressure (e.g., “*Too many things to do at once*”), assorted annoyance (e.g., “*Having your contributions overlooked*”), general social mistreatment (e.g., “*Social isolation*”), romantic problems (e.g., “*Conflicts with boyfriend/girlfriend/spouse*”), and friendship problems (e.g., “*Conflicts with friends*”). Participants reported the extent to which they experienced each event in the past month on a 4-point Likert-type scale, ranging from 1 (*not at all part of my life*) to 4 (*very much part of my life*). The full 49-teim scale can measure a single construct called “hassles” [43]. Scores of all the items was averaged (range: 1–4) to indicate the extent university students experienced stressful events (Cronbach’s alpha was 0.88 in the current sample).

#### 2.2.2. The Big Five Personality Traits

The Big Five Inventory (BFI) [44] assessed the Big Five dimensions of personality. This questionnaire consisted of 44 short-phrase items scored on a 5-point Likert-type scale that ranges from 1 (*strongly disagree*) to 5 (*strongly agree*). The Big Five personality traits were indicated by the average scores (range: 1–5) for the relevant items in the subscales of Extraversion (8 items), Agreeableness (9 items), Conscientiousness (9 items), Neuroticism (8 items), and Openness (10 items), with acceptable-to-good internal consistencies (Cronbach’s alphas were 0.87, 0.71, 0.77, 0.78, and 0.79, respectively) in the present study. 

#### 2.2.3. Stress Mindset

The Chinese Making Sense of Adversity Scale (CMSAS) [45] was developed to measure how university students make sense of stress, and it was recently used in Chen and Qu’s [16,25] studies to measure stress-is-a-challenge and stress-is-a-threat mindsets. A stress-is-a-challenge mindset was measured by 8 items (e.g., “*Stress provides a good opportunity for learning*”), and a stress-is-a-threat mindset was measured by 4 items (e.g., “*Stress means the end of world and I am not able to resolve it*”), on a 4-point Likert-type scale ranging from 1 (*totally disagree*) to 4 (*totally agree*). Scores of all items in each subscale were averaged (range: 1–4) to indicate the extent students believe that stress is a challenge or a threat in general. The Cronbach’s alphas were 0.84 and 0.70 for the challenge and threat subscales, respectively, in our sample.

#### 2.2.4. Coping Flexibility

Coping flexibility was measured by Kato’s [17] Coping Flexibility Scale (CFS), with 8 positively keyed items (e.g., “*If a stressful situation has not improved, I use other ways to cope with the situation*”) and 2 negatively keyed items (e.g., “*I only use certain ways to cope with stress*”), which require reversed scoring. Five items measured evaluation coping (e.g., “*I am aware of how successful or unsuccessful my attempts to cope with stress have been*”), and the other five measured adaptive coping (e.g., “*When I haven’t coped with a stressful situation well, I use other ways to cope with that situation*”). Participants reported how much each item applied to their lives on a 4-point Likert scale (0 = *not applicable at all*, 1 = *somewhat applicable*, 2 = *applicable*, and 3 = *very applicable*). Scores of all the 10 items were summed to indicate coping flexibility (range: 0–30; Cronbach’s *α* = 0.71 in the current sample), with a higher score indicating greater coping flexibility.

#### 2.2.5. Psychological Distress

The 42-item Depression Anxiety Stress Scale (DASS-42) is a self-reported instrument developed by Lovibond and Lovibond [46] to assess psychological distress indicated by three dimensions, namely depression (14 items; e.g., “*I couldn’t seem to experience any positive feeling at all*”), anxiety (14 items; e.g., “*I felt scared without any good reason*”), and stress (14 items; e.g., “*I was in a state of nervous tension*”). Participants reported the extent to which each statement applied to them on a 4-point Likert scale (0 = *did not apply to me at all*, 1 = *applied to me to some degree, or some of the time*, 2 = *applied to me to a considerable degree, or a good part of time*, and 3 = *applied to me very much, or most of the time*). Scores for depression (*M* = 9.08, *SD* = 7.77), anxiety (*M* = 9.15, *SD* = 6.36), and stress (*M* = 12.7, *SD* = 8.02) were calculated by summing the scores of all relevant items in each subscale (range: 0–42). Given that these three dimensions of psychological distress were highly correlated with one another in this study (depression and anxiety: *r* = 0.60, *p* < 0.001; depression and stress: *r* = 0.63, *p* < 0.001; anxiety and stress: *r* = 0.68, *p* < 0.001), we used the total score of all the 42 items to indicate psychological distress (range: 0–126; Cronbach’s *α* = 0 .94 in the current sample). The cut-off score of 60 was used as an indicator of severe psychological distress to identify the prevalence of psychological distress in the current non-clinical sample of university students in Singapore. In total, 10.0% (*n* = 26) of these students experienced severe psychological distress (scored 60 or higher, *M* = 69.7, *SD* = 9.47), whereas the majority (90.0%) lied in the range from normal or mild to moderate (scored 0–59, M = 26.6, SD = 14.7).

### 2.3. Data Analysis

Pearson correlation analysis was used to examine the bivariate correlations among study variables. Analysis of variance (ANOVA) was conducted to test the gender and age differences in all the variables. A series of path analyses was performed using Mplus version 7.31 [47] to examine the proposed mediation models. We used the root mean square error of approximation (RMSEA) and the standardized root mean square residual (SRMR) values below 0.08, as well as the comparative fit index (CFI) and the Tucker–Lewis index (TLI) values above 0.95 to indicate good model fit. Chi-square values were presented for completeness’s sake.

## 3. Results

### 3.1. Preliminary Analysis

Table 1 presents the descriptive statistics and bivariate correlations among the main variables. As expected, Neuroticism was positively associated with perceived stressful experiences, a stress-is-a-threat mindset, and psychological distress but negatively associated with a stress-is-a-challenge mindset and coping flexibility. Conscientiousness, Agreeableness, and Extraversion were all positively correlated with a stress-is-a-challenge mindset and coping flexibility and negatively correlated with a stress-is-a-threat mindset but not related to perceived stressful experiences. Openness only showed a correlation with greater coping flexibility but not with any other variables. ANOVAs yielded a non-significant main effect of age, non-significant main effect of gender, and non-significant interaction effect between age and gender on all the main variables (*ps* > 0.10). 

### 3.2. Roles of Stress Mindset and Coping Flexibility in the Association between Stressful Experiences and Psychological Distress

**Figure 1:**
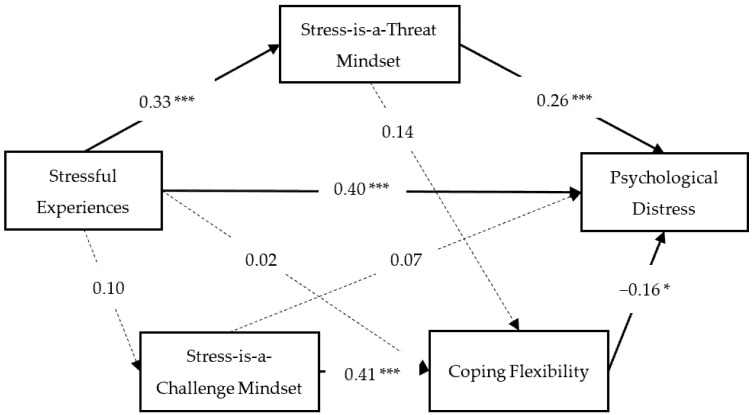
The dual-pathway model of stress coping. Standardized coefficients are presented. Non-significant paths are denoted using dashed lines. Gender (dummy coded as female: 1 = female, 0 = male) was controlled for in this model. *** *p* < 0.001; * *p* < 0.05.

We firstly examined the roles of stress mindset and coping flexibility in the relationship between stressful experiences and psychological distress. As illustrated in Figure 1, a dual-pathway model of stress coping was discovered, with a good model fit, *χ*^2^(1) = 0.85, *p* = 0.36, CFI = 0.99, TLI = 1.01, SRMR = 0.018, RMSEA < 0.001 (90% CI = [0, 0.12]). Stressful experiences were directly associated with greater psychological distress, and this relationship was mediated by a stress-is-a-threat mindset (indirect effect: *β* = 0.30, *SE* = 0.10, *p* = 0.003). We named this pathway a *stress–threat–distress* pathway. 

Meanwhile, a stress-is-a-challenge mindset and coping flexibility, without being influenced by stressful experiences, formed a second pathway to counteract the impact of stressful experiences on psychological distress. A stress-is-a-challenge mindset was related to greater coping flexibility, which then was associated with a lower level of psychological distress (indirect effect: *β* = −0.15, *SE* = 0.07, *p* = 0.03). We named this second pathway a *challenge–flexibility–enhancement* pathway. These variables accounted for 33.8% of the variance in psychological stress (*p* < 0.001).

### 3.3. Relations of the Big Five to the Two Pathways of Stress Coping

We further examined whether the Big Five personality traits were differentially associated with the two pathways of stress coping. Given that the two pathways were somewhat independent, we examined how the Big Five were related to each of the two pathways, respectively. 

**Figure 2:**
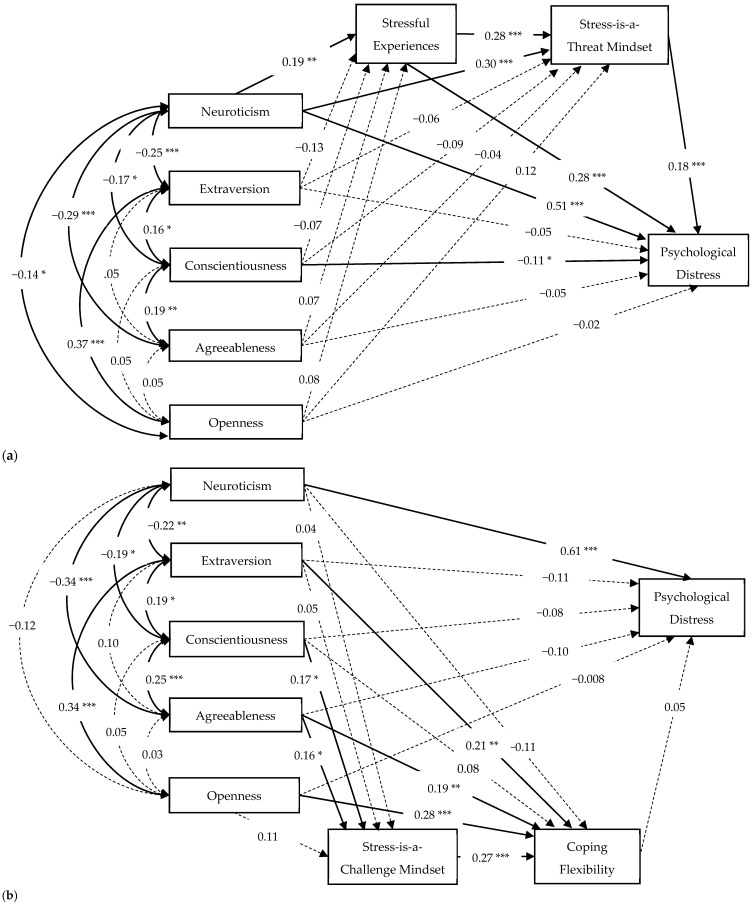
(**a**) The Big Five to the stress–threat–distress pathway. (**b**) The Big Five to the challenge–flexibility–enhancement pathway. Standardized coefficients are presented. Non-significant paths are denoted using dashed lines. *** *p* < 0.001; ** *p* < 0.01; * *p* < 0.05.

Firstly, the relations of the Big Five to the stress–threat–distress pathway were examined. We found that only Neuroticism, but not the other four traits, was significantly related to the stress–threat–distress pathway (see Figure 2a). The model obtained a good model fit, *χ*^2^(7) = 9.78, *p* = 0.20, CFI = 0.98, TLI = 0.94, SRMR = 0.038, RMSEA = 0.049 (90% CI = [0, 0.11]). Neuroticism had a direct effect on psychological distress, and this relationship was mediated by perceived stressful experiences (indirect effect: *β* = 0.05, *SE* = 0.02, *p* = 0.014) and a stress-is-a-threat mindset (indirect effect: *β* = 0.05, *SE* = 0.02, *p* = 0.005). Conscientiousness was associated with a lower level of psychological distress in this model, without being mediated by perceived stressful experiences or a stress-is-a-threat mindset. Together, the Big Five, stressful experiences, and a stress-is-a-threat mindset explained a total of 46.6% of the variance in psychological distress (*p* < 0.001).

Next, the associations of the Big Five with the challenge–flexibility–enhancement pathway were tested. We found that Extraversion, Conscientiousness, Agreeableness, and Openness were associated (either directly or indirectly) with coping flexibility, whereas Neuroticism did not show any relations to a stress-is-a-challenge mindset or coping flexibility (see Figure 2b). The model exhibited a good fit, *χ*^2^(1) = 0.59, *p* = 0.44, CFI = 0.99, TLI = 1.04, SRMR = 0.010, RMSEA < 0.001 (90% CI = [0, 0.19]). Extraversion, Agreeableness, and Openness were directly related to greater coping flexibility, but these relationships were not mediated by a stress-is-a-challenge mindset (indirect effects: Extraversion and Openness, *ps* > 0.10; Agreeableness, *β* = 0.04, *SE* = 0.02, *p* = 0.072). Conscientiousness was positively related to a stress-is-a-challenge mindset, which was then associated with greater coping flexibility (indirect effect: *β* = 0.05, *SE* = 0.02, *p* = 0.048). Possibly due to the strong influence of Neuroticism on emotional symptoms, coping flexibility was not related to psychological distress in this model. Together, the Big Five, a stress-is-a-challenge mindset, and coping flexibility explained a total of 46.4% of the variance in psychological distress (*p* < 0.001).

**Figure 3:**
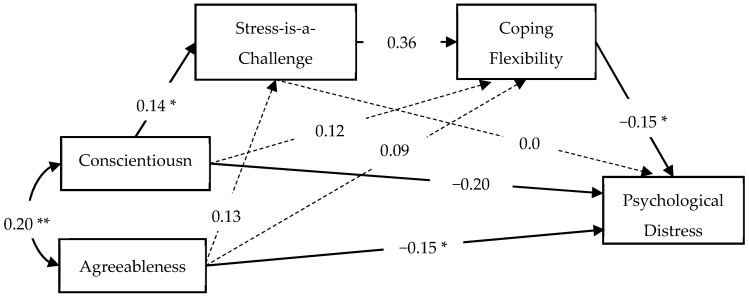
Conscientiousness and the challenge–flexibility–enhancement pathway. Standardized coefficients are presented. Non-significant paths are denoted using dashed lines. ** *p* < 0.01; * *p* < 0.05.

Finally, we conducted a sensitivity analysis to examine whether the association between coping flexibility and psychological distress would become significant when Neuroticism was excluded from the model. Given that only Conscientiousness and Agreeableness were associated with a stress-is-a-challenge mindset in the model, we only included these two personality traits in the sensitivity analysis. A good model fit was obtained, *χ*^2^(2) = 0.60, *p* = 0.74, CFI = 0.99, TLI = 1.12, SRMR = 0.012, RMSEA < 0.001, 90% CI = [0, 0.094]. As expected, the relation of coping flexibility to a lower level of psychological distress became significant (*β* = −0.15, *SE* = 0.07, *p* = 0.038). Moreover, as illustrated in Figure 3, a stress-is-a-challenge mindset mediated the positive association between Conscientiousness and coping flexibility (indirect effect: *β* = 0.05, *SE* = 0.02, *p* = 0.045). Agreeableness was directly related to greater coping flexibility, but this relationship was not mediated by a stress-is-a-challenge mindset (indirect effect: *β* = 0.046, *SE* = 0.03, *p* = 0.072). Coping flexibility acted as the secondary mediator to further mediate the relation of a stress-is-a-challenge mindset to a lower level of psychological distress (indirect effect: *β* = −0.05, *SE* = 0.02, *p* = 0.048). In total, Conscientiousness, Agreeableness, stress-is-a-challenge mindset, and coping flexibility explained 12.9% of the variance in psychological distress.

## 4. Discussion

The current research firstly discovered a dual-pathway model of stress coping with a focus on the roles of stress mindset and coping flexibility in the association between stressful experiences and psychological distress. Furthermore, we found that the Big Five dimensions of personality worked as the risk or protective factors for psychological distress through these two pathways. Neuroticism was associated with the stress–threat–distress pathway, with perceived stressful experiences and a stress-is-a-threat mindset mediating the association between Neuroticism and greater psychological distress. Conscientiousness was associated with the challenge–flexibility–enhancement pathway, with a stress-is-a-challenge mindset and coping flexibility mediating the relation of Conscientiousness to a lower level of psychological distress. Extraversion, Agreeableness, and Openness were directly associated with greater coping flexibility. 

### 4.1. The Two Pathways of Stress Coping: The Roles of Stress Mindset and Coping Flexibility

The dual-pathway model of stress coping established in the current research illustrates how different stress mindsets work with coping flexibility to influence psychological distress. This dual-pathway model with a focus on coping flexibility not only somewhat aligns with but also extends Chen and Qu’s [16] previous dual-pathway model of stress coping. The prior model demonstrated that stressful experiences are related to a stress-is-a-threat mindset, which is associated with an avoidance coping pattern and then contributes to more depressive symptoms, whilst a stress-is-a-challenge mindset is not influenced by stressful experiences but associated with an approach coping pattern, which is then related to fewer depressive symptoms. The current model directly addressed the role of coping flexibility during stress coping.

On the one hand, the stress–threat–distress pathway works as a reactive mechanism associated with more emotional symptoms. This pathway supports the transactional model of stress coping, which demonstrates that interpreting stress as threatening accounts for the impacts of stressful experiences on negative emotions [5,6]. When people experience more stressful events, they may tend to interpret stress as a threat and damage [48] and experience more emotional symptoms such as anxiety, anger, and depression [49]. Exposure to stressful life events or daily hassles and holding a stress-is-threat mindset are risk factors for mental health issues [12,16].

On the other hand, the challenge–flexibility–enhancement pathway acts as a proactive mechanism to counteract the negative impacts of stressful experiences on psychological well-being. A stress-is-a-challenge mindset and coping flexibility can serve as individual-level protective factors to reduce psychological distress, without being influenced by perceived stressful experiences. We argued that a stress-is-a-challenge mindset is associated with greater coping flexibility. The prior studies showed that a challenge state in a specific situation facilitates effective attentional processes and positive affect [50]; and furthermore, adopting an enhancing/challenge mindset (to interpret stress as an opportunity for personal growth and gain in general) is positively associated with situation-strategy fitness, approach coping strategies, cognitive flexibility, and positive affect across different types of stressful situations [10,11,16,25]. These processes can promote people’s ability to evaluate coping strategies, terminate the ineffective ones, and adopt the effective ones to meet different situational demands. Indeed, interventions that aimed to boost a stress-is-a-challenge mindset have shown effectiveness in preventing the decrease in coping flexibility under stress [25]. 

Additionally, the positive relation of coping flexibility to a lower level of psychological distress found in the current sample of university students in Singapore supports the prior research conducted in other countries, which showed that having greater coping flexibility can reduce emotional symptoms such as depression and anxiety in both nonclinical samples [19] and clinical samples [15,51,52]. This challenge–flexibility–enhancement pathway enriches the literature on regulatory flexibility and stress mindset by highlighting the positive relation of a stress-is-a-challenge mindset to coping flexibility and the protective role of a stress-is-a-challenge mindset and coping flexibility in mental health.

### 4.2. The Big Five and the Two Pathways

As a novelty, the current research revealed the mediating roles of different stress mindsets and coping flexibility in the differential relations of the Big Five personality traits to psychological distress. People higher on different personality traits tend to tap into different stress coping pathways. 

Neuroticism is related to the stress–threat–distress pathway. We found that perceiving more experiences of stressful events and holding a stress-is-a-threat mindset account for the relation of Neuroticism to greater psychological distress. This finding is in line with the literature on the mediating role of stressful experiences and negative stress appraisal in the association between Neuroticism and emotional symptoms [3,4,39,53,54,55]. People higher on Neuroticism tend to report more stressful experiences and exaggerate the threat posed by stressful events, both of which intensify their psychological distress such as depressive symptoms and anxiety. When overwhelmed by negative thoughts, negative emotions, and avoidance-motivated behavior, high-Neuroticism people may not be able to engage in any proactive or approach-motivated responses to deal with stress. 

It is noteworthy that, despite the large impact of Neuroticism on emotional symptoms through the stress–threat–distress pathway, Conscientiousness still showed a significant relation to a lower level of psychological distress in the same model. This finding aligns with the protective role of Conscientiousness in psychological well-being demonstrated in the literature [4]. Previous work has shown that Conscientiousness is associated with greater emotional recovery from negative stimuli [56], and its protective role can be accounted for by its relations to positive stress appraisal and approach-motivated coping strategies [4]. We took a further step to reveal that the relation of Conscientiousness to less psychological distress can be explained by the challenge–flexibility–enhancement pathway, with a stress-is-a-challenge mindset and coping flexibility as the mediators. People higher on Conscientiousness are more likely to adopt a stress-is-a-challenge mindset, which then plays a positive role in coping flexibility. Conscientious individuals are usually achievement-oriented and have positive appraisal of a stressful event, so they may have shaped a stress-is-a-challenge mindset to acknowledge the opportunities for personal growth and gain inherent in stress, independent of any situation. While they are cautiously exploring the environment and carefully planning for actions, having a stress-is-a-challenge mindset can facilitate them to actively reflect on their current coping strategies, relinquish the ineffective strategies and adopt alternative ones from their wide collection of approach coping strategies, based on the situational characteristics. Eventually, their high coping effectiveness across situations can enhance their psychological well-being despite the exposure to adversity.

Agreeableness, Extraversion, and Openness were found in this study to be directly associated with greater coping flexibility, regardless of people’s stress mindset. People high on Agreeableness tend to adopt a prosocial orientation towards others and the environment, so they can obtain more resources from their environment and interpersonal relationships to cope with various stressors [37]. With effective evaluations of changing situations and great compliance with the environment, agreeable individuals may be able to modify coping strategies efficiently so as to meet the changing situational demands. People high on Extraversion usually have great sociability, which enables them to attain more resources and build up a collection of functional coping strategies to deal with diverse stressful events [4,38]. Their assertiveness and high activity may also facilitate them to efficiently modify coping strategies according to the situational characteristics. People who are open to new experiences often have the curiosity to explore different coping strategies to deal with a situation, and they also have the creativity to utilize and modify these strategies in a flexible manner. Therefore, these three personality traits can directly contribute to greater coping flexibility.

To sum up, whilst Neuroticism is usually a risk factor for mental health issues, other personality traits such as Conscientiousness, Agreeableness, Extraversion, and Openness can be resilience factors for maintaining or promoting mental health [57,58], with a positive stress mindset and/or coping flexibility as the key.

### 4.3. Implications

The present research has made some contributions to the field. The dual-pathway model of stress coping has advanced our understanding of how stress mindset and coping flexibility work with each other to influence psychological well-being. Our findings have extended previous research on stress coping by revealing the mediating role of coping flexibility in the association between a stress-is-a-challenge mindset and fewer emotional symptoms. Moreover, this study fills the gaps in understanding how the Big Five personality traits relate to coping flexibility. By introducing coping flexibility to the framework on personality and stress coping, our findings add to the literature on the mechanism by which personality traits influence psychological health. 

Our findings also shed light on future prevention or intervention programs to reduce anxiety and depressive symptoms. The current work suggests that a stress-is-a-challenge mindset and coping flexibility are individual-level protective factors for reducing psychological distress. These two factors can be integrated to future interventions to promote *resilience*, which refers to the phenomenon that some individuals are able to maintain mental health instead of becoming mentally ill or even achieve more adaptive psychological outcomes after experiencing adversity [57,59]. Previous research deemed positive interpretational style and adaptive coping as important determinants of resilience [57,58,59,60]. Our work further highlights the critical role of a stress-is-a-challenge mindset and coping flexibility in fostering resilience. Given that our model was established in a non-clinical sample, the findings suggest that, boosting a challenge mindset to focus on the enhancing outcomes of stress and training the ability to evaluate and modify coping strategies according to the situational characteristics can prevent typically developing individuals from emotional symptoms during or after the exposure of daily hassles. For example, the Cognitive Bias Modification of Interpretation (CBM-I) program is an effective prevention or intervention program to reduce attentional and interpretational biases, increase positive stress interpretation of real-life stressors, and reduce emotional symptoms [61,62]. CBM-I can also enhance a stress-is-a-challenge mindset, which then prevents the decrease in coping flexibility under stress [25]. Moreover, based on the recent finding that cognitive remediation therapy (CRT) can promote a positive attitude and coping flexibility in clinical samples such as those with anorexia nervosa [63], we believe that the challenge–flexibility–enhancement pathway can also be used to guide treatment for patients with emotional disorders such as depression and anxiety, to facilitate a more rapid and successful recovery. However, this suggestion requires further empirical examinations because our findings were based on a non-clinical sample of university students in Singapore.

Furthermore, the mechanism through which the Big Five dimensions of personality influence psychological distress recommend that personality traits can be taken into account when deigning prevention or intervention programs. For instance, the relation of Neuroticism to the stress–threat–distress pathway and the relation of Conscientiousness to the challenge–flexibility–enhancement pathway indicates the importance of training high-Neuroticism people to break the pre-established belief about the debilitating nature of stress, to practice a positive mindset to focus on the enhancing outcomes of stress, to learn new functional coping strategies to replace the dysfunction ones, and to improve coping flexibility. Last but not the least, findings on the direct or indirect relations of the Big Five to coping flexibility suggest that coping flexibility may be improved by training people’s planning skills (related to Conscientiousness), sociability (related to Extraversion), prosocial orientation and compliance with environment (related to Agreeableness), as well as cognitive flexibility and creativity (related to Openness). 

In sum, psychotherapy that aims at modifying negative stress interpretation, boosting positive interpretation, improving coping flexibility, and even training personal strength related to Conscientiousness, Agreeableness, Extraversion, and Openness may be an effective treatment (in addition to pharmacotherapy) for emotional disorders such as depression and anxiety. 

### 4.4. Limitations and Future Directions

The present research has several limitations that can provide insights to future research. The first limitation had to do with the self-report measures, which may not have accurately captured how participants behave in the actual situation. Future studies will benefit from including implicit measures or biomarkers to measure participants’ motivational and emotional states and using observational or behavioral measures to assess coping flexibility. Second, due to the small sample size, we could not further investigate the combined effects of different personality traits on stress coping. For instance, the combination of high Neuroticism and low Conscientiousness predicted high levels of perceived stress, dysfunctional coping patterns, and poorer health [64,65,66]. Thus, future research should investigate the interaction effects between Neuroticism and Conscientiousness or other combinations on coping flexibility. Third, the dual-pathway model established in a cross-sectional design only illustrates the relationships among the main variables at a given point in time. Therefore, it is necessary to use an experimental study or an intervention study with a longitudinal design to further examine the long-term effect of trained stress mindset and coping flexibility on psychological well-being. Relatedly, as discussed in the previous sub-section, it is of great significance to replicate the model in other samples, such as populations diagnosed with emotional disorders and patients with chronic conditions, so as to inform future treatment for depression and anxiety via psychotherapy.

## 5. Conclusions

The current research established a dual-pathway model to illustrate how stress mindset and coping flexibility influence the impacts of stressful experiences on psychological distress. We found that a stress-is-a-threat mindset is a risk factor that carries the negative impact of stressful experiences on psychological distress; conversely, a stress-is-a-challenge mindset is a protective factor for psychological well-being because of its relation to greater coping flexibility. Moreover, the two pathways, namely the stress–threat–distress pathway and the challenge–flexibility–enhancement pathway, can explain how the Big Five personality traits influence psychological distress. Stressful experiences and a stress-is-a-threat mindset mediate the impacts of Neuroticism on psychological distress. A stress-is-a-challenge mindset and coping flexibility mediate the relation of Conscientiousness to a lower level of psychological distress. Extraversion, Agreeableness, and Openness are all associated with greater coping flexibility, which has been acknowledged as a strong predictor of fewer emotional symptoms and better mental health. These findings advance our understanding of the complex mechanism through which psychological distress such as depression, anxiety, and stress may be intensified or alleviated and inform future intervention programs to promote mental health. 

## Figures and Tables

**Table 1:** Descriptive statistics and bivariate correlations among the main variables (*N* = 260).

	1	2	3	4	5	6	7	8	9	10	11	12
1. Extraversion	-	0.11	0.17 **	−0.26 ***	0.38 ***	−0.09	0.16 **	−0.14 *	0.37 ***	−0.23 **	−0.07	0.10
2. Agreeableness		-	0.22 ***	−0.25 ***	0.08	−0.14	0.15 *	−0.12 *	0.21 ***	−0.23 **	0.04	0.05
3. Conscientiousness			-	−0.20 **	0.06	−0.12	0.17 **	−0.17 **	0.22 ***	−0.23 ***	−0.01	0.15 *
4. Neuroticism				-	−0.17 **	0.30 ***	−0.16 *	0.39 ***	−0.29 ***	0.64 ***	0.10	0.06
5. Openness					-	−0.03	0.11	0.05	0.38 ***	−0.08	−0.07	0.02
6. Stressful Experiences						-	0.10	0.33 ***	−0.001	0.48 ***	−0.24	0.12
7. Challenge Mindset							-	−0.06	0.36 ***	−0.04	0.08	0.02
8. Threat Mindset								-	−0.11	0.52 ***	0.003	0.13
9. Coping Flexibility									-	−0.23 ***	−0.005	0.07
10. Distress										-	−0.001	0.07
11. Age											-	−0.34 ***
12. Gender (Female)												-
*M*	2.93	3.67	3.18	2.95	3.34	1.89	3.16	1.52	17.8	30.7	21.4	60.4%
*SD*	0.77	0.54	0.61	0.68	0.59	0.31	0.49	0.46	4.18	19.3	1.70	-
*Min*	1.25	1.78	1.67	1.00	1.60	1.08	1.63	1.00	6	0	19	-
*Max*	5.00	5.00	4.78	5.00	4.80	2.96	4.00	4.00	30	90	26	-

*** *p* < 0.001; ** *p* < 0.01; * *p* < 0.05.

## Data Availability

The raw data supporting the conclusions of this article will be made available by the authors upon reasonable request.
